# Efficient Photochemical Vapor Generation from Low Concentration Formic Acid Media

**DOI:** 10.1021/acs.analchem.3c04472

**Published:** 2024-01-06

**Authors:** Eva Jeníková, Jaromír Vyhnanovský, Karolína Hašlová, Ralph E. Sturgeon, Stanislav Musil

**Affiliations:** † Institute of Analytical Chemistry of the Czech Academy of Sciences, Veveří 97, Brno 602 00, Czech Republic; ‡ Faculty of Science, Charles University, Hlavova 8, Prague 128 43, Czech Republic; § National Research Council of Canada, 1200 Montreal Road, Ottawa, Ontario K1A 0R6, Canada

## Abstract

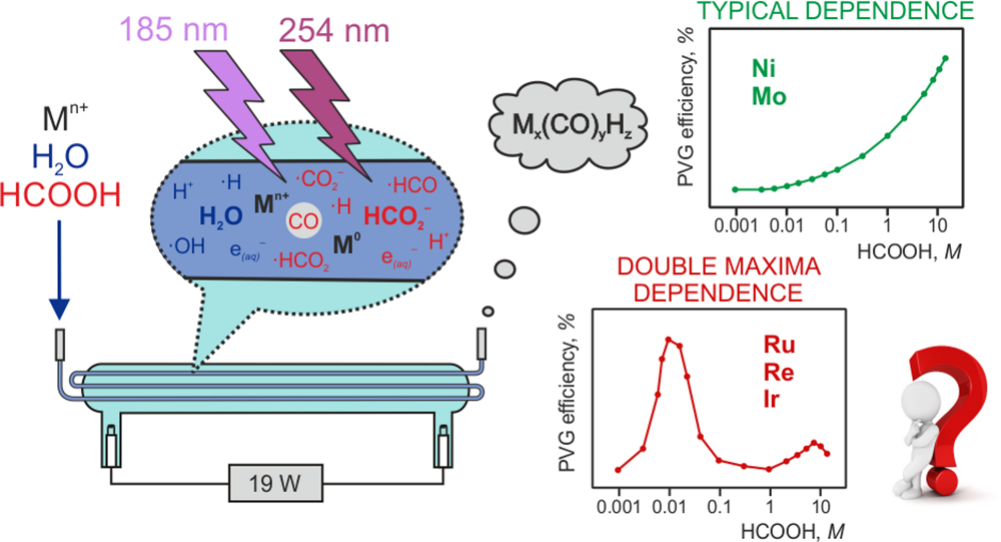

Herein, we report on surprisingly efficient photochemical vapor generation (PVG) of Ru, Re, and especially Ir, achieved from very dilute HCOOH media employing a thin-film flow-through photoreactor operated in flow injection mode. In the absence of added metal ion sensitizers, efficiencies near 20% for Ir and approximately 0.06% for Ru and Re occur in a narrow range of HCOOH concentrations (around 0.01 M), significantly higher than previously reported from conventionally optimized HCOOH concentrations (1–20 M). A substantial enhancement in efficiency, to around 9 and 1.5%, could be realized for Ru and Re, respectively, when 0.005 M HCOONa served as the PVG medium. The addition of metal ion sensitizers (particularly Cd^2+^ and Co^2+^) to 0.01 M HCOOH significantly enhanced PVG efficiencies to 17, 2.2, and 81% for Ru, Re, and Ir, respectively. Possible mechanistic aspects occurring in dilute HCOOH media are discussed, wherein this phenomenon is attributed to the action of 185 nm radiation available in the thin-film flow-through photoreactor. An extended study of PVG of Fe, Co, Ni, As, Se, Mo, Rh, Te, W, and Bi from both dilute HCOOH and CH_3_COOH was undertaken, and several elements for which a similar phenomenon appears were identified (i.e., Co, As, Se, Te, and Bi). Although use of dilute HCOOH media is attractive for practical analytical applications employing PVG, it is less tolerant toward dissolved gases and interferents in the liquid phase due to the likely lower concentrations of free radicals and aquated electrons required for analyte ion reduction and product synthesis.

## Introduction

Photochemical vapor generation (PVG) remains an evolving alternative sample introduction technique useful for coupling to all analytical atomic spectrometries.^1^ It offers all the inherent features common to other vapor generation techniques, such as enhanced introduction efficiency in comparison to solution nebulization and separation of analyte from the liquid matrix, thus alleviating possible spectral and nonspectral interferences during detection. The analyte is converted to volatile species following UV irradiation of the liquid photochemical media. To date, mainly low molar mass organic acids, primarily formic and acetic acid, have been employed for this purpose as their photolysis yields strongly reducing radical species (H^•^, R^•^, and CO_2_^•–^) and aquated electrons (e_(aq)_^–^).^1,2^ These subsequently interact with the ionic analytes to ultimately form volatile free atoms (Hg^0^), hydrides, carbonyls, or alkylated derivatives, depending on the element and photochemical medium used. Oxidative conditions have also been advantageously used for the PVG of volatile OsO_4_ from dilute peroxide, nitric acid, or pure water.^3−5^

Simple PVG photoreactors typically comprise a UV source (most often a low-pressure Hg discharge lamp emitting primarily 254 nm radiation) and a conduit of UV transmissible material placed in close proximity to the source to permit irradiation of sample solutions.^6^ Advanced thin-film flow-through photoreactors employ a modified low-pressure Hg discharge lamp wherein the sample is irradiated in a synthetic quartz channel directly immersed in the discharge.^7^ Since UV photons have to pass only the thin synthetic quartz wall of the inner channel, this photoreactor permits ready access to intense 185 nm radiation in addition to 254 nm, efficiently photolyzing the sample without concurrent ozone generation. This thin-film photoreactor is becoming the generator design of choice due to its applicability to many analytes, high photon flux, access to vacuum UV, and short sample irradiation times, resulting in fast sample throughput.

The number of analytes to which PVG can be applied continues to expand and now includes Hg and all elements typically forming volatile hydrides, the halogens, and some 18 transition metals. This more recent expansion was due largely to the use of metal ion sensitizers (especially Cd^2+^,^8−10^ Co^2+^,^11−18^ Cu^2+^,^19−23^ Fe^2+/3+^,^4,24−28^ Ni^2+^,^15^ and V^4+/5+^ (ref (29))) that significantly increase PVG efficiencies or were found indispensable in giving rise to PVG products of several elements. Synergistic effects have been reported for several analytes when a combination of two metal sensitizers is employed, resulting in enhanced efficiencies above that achieved with either one alone, namely, Co^2+^ and Cu^2+^ ions,^30^ Cd^2+^ and Co^2+^ ions,^31−34^ and Mn^2+^ and Fe^2+^ ions.^35^ Apart from the effect of added metal ion sensitizers, the analyte response optimization strategy is mainly focused on the impact of irradiation time (IT), composition of the photochemical medium, and solution pH. HCOOH has been almost exclusively employed as the photochemical medium for PVG of transition metals.^1,2^ Some PVG studies revealed a positive effect of adjusting HCOOH media with hydroxides to yield higher concentrations of HCOO^–^ anion.^34,36−41^ The typically examined HCOOH concentrations range from 1 to 20 M. Since the dependences often exhibit sharply decreasing PVG efficiencies while lowering the HCOOH concentration, a zero concentration of HCOOH (pure water) was only occasionally examined to verify that no PVG action arose from this medium;^23,36,37,42^ consequently, the concentration region close to zero was logically not investigated.

Herein, we highlight a remarkable observation encountered during our follow-up studies of PVG of Ru, Re, and Ir using a thin-film flow-through photoreactor.^33,34,43^ Surprisingly higher PVG efficiencies can be obtained from very dilute HCOOH media compared to conventionally optimized HCOOH concentrations (1–20 M). This phenomenon was studied in detail for Ru, Re, and Ir by investigating the effects of HCOOH and HCOONa concentrations, irradiation times, the presence of metal ion sensitizers, dissolved gases, and availability of UV wavelengths. Some mechanistic aspects are discussed. Additionally, investigation of PVG from dilute HCOOH and CH_3_COOH media was undertaken with the aim of identifying other elements for which the same phenomenon may arise.

## Experimental Section

### Chemicals

Formic acid (98%, ≈26 M, p.a., Lach-Ner, Czech Republic) was used for the formulation of photochemical media of various molarities (M, i.e., mol L^–1^). Nitric acid (≥65%, semiconductor grade) and sodium formate were sourced from Sigma-Aldrich; acetic acid (99.8%, p.a.) was from Lach-Ner. Deionized water (DIW, <0.2 μS cm^–1^, Ultrapur, Watrex) was used for the preparation of all photochemical media. DIW was also used for the serial dilution of all stock solutions (no acids added) with the exception of mixed working standards that were prepared with the required molarity of HCOOH to match that of the tested photochemical medium. The specification of individual analytical standards and compounds used as potential metal ion sensitizers is given in the Supporting Information. Mixed standards used for PVG contained no more than three analytes (e.g., Ru^3+^, Re^7+^, and Ir^3+^; Ni^2+^ and Mo^6+^; Fe^3+^, Co^2+^, and W^6+^, etc.) prepared in the photochemical medium in addition to possibly being spiked with various metal ion sensitizers.

For some experiments, the photochemical media were saturated with Ar (99.996% purity), O_2_ (99.5%), or CO (99.9%) obtained from SIAD Ltd. (Czech Republic).

### Instrumentation

The PVG system, as described in our recent studies on PVG of Ru and Ir,^33,34^ comprises a 19W thin-film flow-through photoreactor (Jitian Instruments Co., China), based on a flow injection (FI) mode of operation. The outlet of a gas–liquid separator was coupled to ICPMS via an ultrahigh matrix introduction port located downstream of a Scott double-pass spray chamber, which was concurrently fed with a pneumatically nebulized (PN) stream of carrier liquid (2% (m/v) HNO_3_) containing an internal standard (see Figure S1 and the Supporting Information for the scheme and details of the entire arrangement). Detection was performed using an Agilent 8900 triple quadrupole ICPMS operating in time-resolved analysis and single quadrupole modes. Optimal plasma settings for PVG measurements and monitored analyte and internal standard isotopes are summarized in Table S1.

### Procedure, Data Evaluation, and Conventions

A standard prepared in the photochemical medium (possibly spiked with selected sensitizers) was manually injected into the carrier stream at the beginning of a PVG cycle of recording signal intensities. Integration was stopped after the transient signals returned to the baseline. The peak area (counts) of the FI transients, normalized to the averaged signal from the internal standard continuously admitted by PN over the same time window, was employed as a measure of analyte response and used for the evaluation of the overall PVG efficiency. PVG efficiency was the main parameter of interest and is displayed in the figures and tables. Overall PVG efficiency is defined as the fraction of analyte converted to volatile species, released to the gas phase, and transported to the plasma. It was estimated for individual PVG conditions (in accordance with our previously published procedure) as the product of a sensitivity enhancement factor and absolute PN efficiency.^33,34^ The latter is defined as the fraction of analyte nebulized into the spray chamber and transported to the plasma, as determined using a dynamic mass flow approach.^44^ PN efficiency was typically around 7.6% and was periodically determined under optimal settings of the ICPMS (Table S1) when coupled with PVG. The enhancement factor was determined as the ratio of the peak area sensitivity (in counts μg^–1^) obtained with FI-PVG sample introduction to that arising from FI-PN during their concurrent operation, i.e., both measured under exactly the same plasma conditions.^10,11,33−35^ Analyte concentrations employed for FI-PVG differed throughout this study depending on the actual ICPMS peak area sensitivity driven by the PVG efficiency. The typical concentration employed for FI-PN was 1 μg L^–1^ prepared in 2% (m/v) HNO_3_. The concentrations used for FI-PVG are summarized for individual dependences in the relevant figure captions. Each determined PVG efficiency is presented in the figures and tables as the average evaluated from at least three peak area replicates with an associated uncertainty given as ± one combined standard deviation (SD).

## Results and Discussion

Follow-up experiments to our previous studies on PVG of Ru,^33^ Re,^43^ and Ir^34^ fortuitously revealed interesting and repeatable conditions that resulted in surprisingly “high” PVG efficiencies for these metals from aqueous solutions containing only traces of HCOOH (around 0.01 M HCOOH). These results are to be contrasted with typical conditions frequently employing single to tens of molar levels of HCOOH in which a clear maximum in response (i.e., PVG efficiency) is attained or a plateau is reached. These tests were originally conducted to confirm that PVG undertaken in only DIW would generate no response, but, surprisingly, this photochemical medium also produced analyte signals, subsequently shown to be due to the presence of trace levels of HCOOH derived from the added spikes of standard solutions of the analyte elements prepared in 1 M HCOOH. If traces of HCOOH introduced by the prepared standards were avoided, by a serial dilution of all stock analyte standard solutions with only DIW, absolutely no response from these elements was detected. The range of suitable HCOOH concentrations in which PVG efficiency was markedly enhanced appeared to be very narrow, which may partially account for why it has not been earlier identified in all the recent papers devoted to PVG of Ru, Re, and Ir,^23,31−34,43^ where the dependences of analytical response on HCOOH concentration often exhibited sharply decreasing PVG efficiencies when lowering the HCOOH concentration and the low concentrations were logically not thoroughly investigated.

### PVG from Dilute HCOOH Media

**Figure 1:**
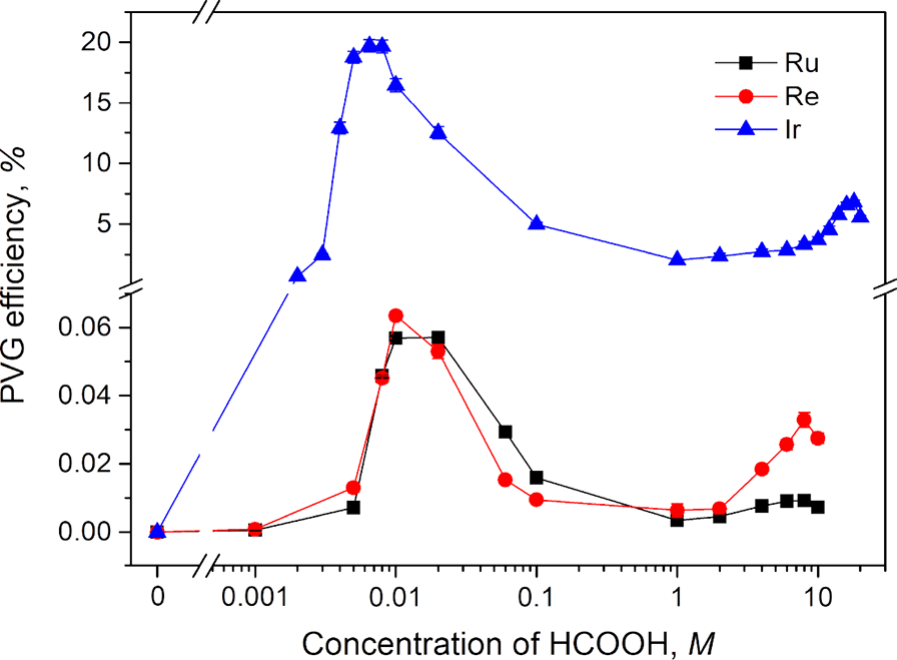
Influence of HCOOH concentration at a sample flow rate 2 mL min^–1^ (IT = 22 s) on PVG efficiency of Ru, Re, and Ir determined with 40 μg L^–1^ Ru and Re and 0.2 μg L^–1^ Ir. Note that the range 0.0005 to 30 M is presented on a logarithmic scale.

The influence of the HCOOH concentration on the PVG of Ru, Re, and Ir was studied first, without added sensitizers, while taking care to account for any HCOOH introduced to the photochemical medium via the spiked analyte standards. A particular focus converged on the narrow range of concentration between 0.001 and 0.1 M HCOOH, but the dependences were also examined at higher concentrations for comparison. The effects on Ru, Re, and Ir at a sample flow rate of 2 mL min^–1^ (IT = 22 s) are displayed in Figure 1. All three dependences exhibit two maxima in PVG efficiency (response), wherein the first narrow maximum for all three analytes occurs around 0.01 M HCOOH and provides significantly higher PVG efficiency than the second maximum occurring in the range 6–18 M, depending on the analyte. PVG efficiency for Ir is surprisingly high, reaching almost 20% at 0.008 M HCOOH and 17% at 0.01 M HCOOH, while those for Ru and Re are more than 2 orders of magnitude lower, i.e., 0.057 and 0.064%, respectively. This poor PVG efficiency throughout the entire range of HCOOH concentrations tested is consistent with all recent reports, highlighting the necessity of addition of Co^2+^ and Cd^2+^ sensitizers to markedly enhance PVG efficiencies of these two metals.^31−33^ Since the PVG efficiencies for Ru and Re were low, care was taken to account for any contribution of cogenerated aerosol to the measured peak area responses. Cesium, added to the injected standards, was used to monitor any aerosol fraction associated with either analyte since no PVG occurs for Cs. The peak area response obtained with FI-PVG in 0.01 M HCOOH was compared to that obtained with FI-PN of standards in 2% HNO_3_ in the same manner as used for estimation of PVG efficiency. The efficiency of Cs introduction was determined to be 2.0 ± 0.2 × 10^–8^%, dispelling the possibility of any contribution to the response arising from physical transport of the analyte elements. All trends in Figure 1 are thus fully attributable to the action of PVG. This is supported by the absence of a PVG response from 0 M HCOOH, i.e., irradiation of a medium of only analyte in DIW (see Figure S2A–C for comparison of the signals from a standard containing 40 μg L^–1^ Ru and Re and 0.1 μg L^–1^ Ir prepared in DIW and typical FI signals from the standard with the same analyte concentrations prepared in 0.01 M HCOOH).

In addition to the HCOOH concentration, PVG efficiency also depends on the sample IT. With the flow-through reactor, IT is inversely proportional to the sample flow rate. Figure S3 illustrates such effects for Ru, Re, and Ir when employing 0.01 M HCOOH as the photochemical medium, selected for study as a reasonable compromise in PVG efficiencies for all three analytes (Figure 1). Distinct maxima in PVG efficiencies occur at 1.5 mL min^–1^ (IT = 29 s) for Ir and 2 mL min^–1^ (IT = 22 s) for Re, whereas a broader plateau of maximum PVG efficiency occurs in the range 2–3 mL min^–1^ for Ru. The decreased PVG efficiencies at lower flow rates (longer ITs) are rather remarkable, declining to zero for Ru and Re at 1 mL min^–1^ (IT = 43 s). Quite the opposite trends occur for Re and Ir when PVG is undertaken with 6 M HCOOH for Re and 10 and 14 M HCOOH for Ir.^34,43^ Sensitivity gradually increases with lower sample flow rates down to 0.5 mL min^–1^ (IT = 86 s). If it is assumed that the same volatile species are produced, irrespective of the concentration of HCOOH, the difference in the effects of IT employing either 0.01 M HCOOH or 6–14 M HCOOH may be attributed to two factors: greater photon penetration depths for both 254 and especially the more energetic 185 nm source radiation (discussed below) as well as the poor (photo)stability of the generated species (presumably metal carbonyls). The dilute medium cannot provide an equivalent UV shadowing effect, with the consequence that the volatile species are more rapidly decomposed in the condensed phase following their generation. At a sample flow rate of 1 mL min^–1^ (IT = 43 s), maximum PVG efficiency for Ir is clearly shifted from 0.008 to 0.02 M HCOOH, whereas those for Ru and Re remain close to zero. This observation supports the impact of the UV shadowing effect (i.e., photon penetration depth).

Whereas dilute HCOOH medium is capable of reducing both Ir^3+^ and Ir^4+^ followed by generation of volatile carbonyl species, no PVG response is observed using dilute CH_3_COOH, as detailed in the Supporting Information and is consistent with the lack of photolytic generation of CO in this medium.^45^

### PVG from Dilute HCOONa Media

The rate of photolysis of HCOOH is related to the degree of dissociation of the acid.^46^ HCOOH is a weak acid with p*K*_a_ around 3.75, which means that at 0.01 M HCOOH, the degree of dissociation to HCOO^–^ is approximately 13%. The impact of HCOONa (100% dissociation), and thus pH, on the PVG of all three analytes was first investigated by varying its fraction in the photochemical medium when the sum of concentrations of HCOOH and HCOONa was always 0.01 M, based on the observation noted in Figure 1. The dependences of PVG efficiency obtained at a sample flow rate of 2 mL min^–1^ are displayed in Figure S4 where it is evident that PVG efficiency for Ru gradually increased with the fraction of HCOONa in the medium up to around 8% for the neat 0.01 M HCOONa (pH = 7.9). A similar behavior was observed for Re, wherein the highest PVG efficiency (approaching 1%) was attained with 0.01 M HCOONa while the efficiency remained below 0.08% with all the acidic media tested: 0–90% HCOONa fractions (pH = 2.9–4.7). For Ir, the maximum PVG efficiency of 31% was identified using 0.0025 M HCOOH + 0.0075 M HCOONa (pH = 4.2). Surprisingly, higher fractions of HCOONa lead to a serious decrease in PVG efficiency.

**Figure 2:**
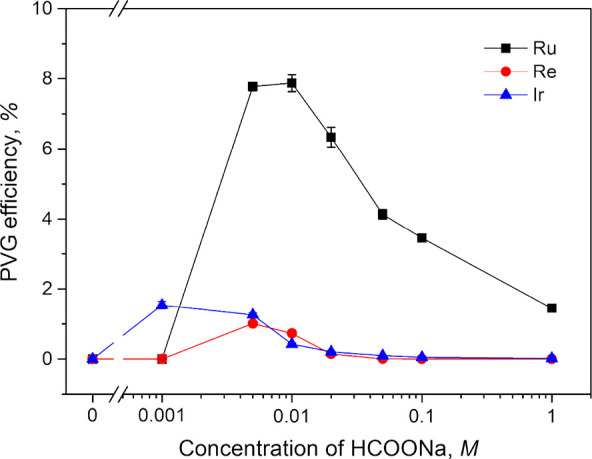
Influence of HCOONa concentration at a sample flow rate 2 mL min^–1^ (IT = 22 s) on PVG efficiency determined with 5 μg L^–1^ Ru and Re and 0.1 μg L^–1^ Ir. The range 0.0005 to 2 M is presented on a logarithmic scale.

Subsequently, the photochemical medium was prepared from only HCOONa (no added HCOOH). Figure 2 shows the effect of HCOONa in the range of 0.001–1 M on PVG at a sample flow rate 2 mL min^–1^. It is evident that maximum PVG efficiency for Ru is reached in the range 0.005–0.01 M HCOONa (pH = 7.7–7.9) and at 0.005 M HCOONa for Re, i.e., at very similar molar concentrations when HCOOH is used (Figure 1). Compared to the response from 0.01 M HCOOH (pH = 2.9), a more than 100-fold enhancement in PVG efficiency was achieved for Ru, while that for Re was around 16-fold (cf. Figure 1). Conversely, PVG efficiency for Ir generated with any concentration of HCOONa was always significantly lower.

A concentration of 0.005 M HCOONa in the photochemical medium was further selected to examine the effect of IT (Figure S5). The optima for Ru and Re appeared at 1.8 and 1.5 mL min^–1^, respectively, corresponding to PVG efficiencies of 9.4 and 1.5%. PVG efficiency for Ir remained below 2% in the range 1.8–3 mL min^–1^, whereas an almost 10-fold enhancement was identified at 1.3 mL min^–1^. The significant decline in PVG efficiencies at low sample flow rates, similar to that described above in relation to Figure S3, suggests that photodecarbonylation of the generated metal carbonyl species occurs due to excessive UV irradiation. Employing a mixture of 0.0025 M HCOOH + 0.0075 M HCOONa, with which a significant enhancement in PVG was specifically identified for Ir (Figure S4), the maximum PVG efficiencies of 31.7 and 31.0% were reached at sample flow rates 1.5 and 2 mL min^–1^, respectively.

### Impact of Metal Ion Sensitizers

The addition of metal ion sensitizers plays a critical role in enhancing PVG efficiencies for Ru, Re, and Ir from media containing “normal” HCOOH concentrations.^31−34,43^ For analytical purposes, a mixture of 10 mg L^–1^ Co^2+^ and 25 mg L^–1^ Cd^2+^ as sensitizers added to 8 and 4 M HCOOH was found optimal for PVG of Ru and Ir, respectively, providing efficiencies of almost 30 and 90%. Investigation of the effect on PVG of Re has not yet been completed in our laboratory,^43^ but insights may be gleaned from the study by Zhen et al.^32^ These authors employed a stop flow mode of generation and achieved a significant enhancement in PVG efficiency using 5 mg L^–1^ Co^2+^ and 30 mg L^–1^ Cd^2+^ as sensitizers added to a mixture of 20% (v/v) HCOOH (≈5 M) and 15% (v/v) CH_3_COOH (≈2.6 M). The absolute PVG efficiency was, however, not reported.

Using a 0.01 M HCOOH medium, the effect of the individual metal ion sensitizers (Cd^2+^, Co^2+^, Cu^2+^, Fe^2+^, Mn^2+^, and Ni^2+^) on the PVG efficiency of Ru, Re, and Ir was examined (Figure S6A–C). The greatest impacts were observed with the addition of 5–20 mg L^–1^ Cd^2+^ ions for all three analytes. A very similar enhancement was also observed for Co^2+^ and Fe^2+^ on PVG of Ru but at orders of magnitude higher concentrations. The other metal ions had significantly lower or negligible effects on PVG. PVG efficiencies achieved under various experimental conditions with and without the most effective sensitizer(s) are compared in Table 1, including PVG efficiencies reported previously.^33,34^ For the purpose of comparison, PVG conditions for Re reported as optimal by Zhen et al.^32^ were adopted for this work using the FI mode of the photoreactor (1 mL min^–1^); the resulting PVG efficiency is included in Table 1.

**Table 1:** PVG Efficiencies Achieved with Different Concentrations of HCOOH/HCOONa in the Photochemical Media with and without Added Metal Ion Sensitizers

photochemical medium (M)	sample flow rate (mL min^–1^)	sensitizer(s) (mg L^–1^)	PVG efficiency (%)
**Ru**			
HCOOH (0.01)	2		0.057 ± 0.001
HCOOH (8)	2		0.009 ± 0.001
HCOONa (0.005)	1.8		9.4 ± 0.2
HCOOH (0.01)	2	Cd (10)	8.5 ± 0.1
HCOOH (0.01)	1.25–1.5	Co/Cd (10/25)	16.3–16.6
HCOOH (8)	1.4	Co/Cd (10/25)	29.0 ± 0.1^a^
**Re**			
HCOOH (0.01)	2		0.064 ± 0.001
HCOOH (8)	2		0.033 ± 0.002
HCOONa (0.005)	1.5		1.50 ± 0.02
HCOOH (0.01)	2	Cd (5)	2.05 ± 0.04
HCOOH (0.01)	0.75	Co/Cd (10/25)	2.22 ± 0.07
HCOOH/CH_3_COOH (5/2.6)	1	Co/Cd (5/30)	3.1 ± 0.2^b^
**Ir**			
HCOOH (0.01)	1.5		23.8 ± 0.5
HCOOH (0.01)	2		16.5 ± 0.5
HCOOH (18)	2		6.8 ± 0.2
HCOOH/HCOONa (0.0025/0.0075)	1.5–2		31.0–31.7
HCOOH (0.01)	2	Cd (5)	76.2 ± 1.4
HCOOH (0.01)	1–1.5	Co/Cd (10/25)	78.7–80.8
HCOOH (4)	1.5	Co/Cd (10/25)	88.6 ± 0.6^c^

a Taken from ref (33).

b Conditions adopted from ref (32) and used in FI mode of the photoreactor.

c Taken from ref (34).

Additional enhancements may possibly be achieved using other combinations of metal sensitizers. As noted earlier, a strong synergistic effect was identified using a combination of Co^2+^ and Cd^2+^ sensitizers for Ru, Re, and Ir when PVG was conducted with “normal” concentrations of HCOOH.^31−34,43^ In this work, the aim was not to investigate the various combinations of metal ion sensitizers in detail but at a selected 10 mg L^–1^ Co^2+^ and 25 mg L^–1^ Cd^2+^ previously found optimal for PVG of Ru and Ir.^33,34^ This enabled direct comparisons with earlier reported PVG efficiencies. The sample flow rate was optimized with this mixture using 0.01 M HCOOH as the photochemical medium; optimal values are given in Table 1. It is important to note that maximum PVG efficiency is achieved over a broader range of flow rates, i.e., 1.25–1.5 mL min^–1^ for Ru and 1–1.5 mL min^–1^ for Ir. At a flow rate of 0.5 mL min^–1^, no response was detected for Ru and Re while the PVG efficiency for Ir was reduced to 1.9%. These observations further support the suggestion that decomposition of generated volatile species occurs during excessive UV irradiation, although this effect manifests itself at longer ITs than without added sensitizers (cf. Figure S3). The reason may lie with a UV shadowing effect (especially of 185 nm) from added Co^2+^ and Cd^2+^ sensitizers, although absorption of 185 nm has never been conclusively proven because the UV spectra of added sensitizers have typically been limited to 190 nm.^10,33^

A further interesting observation arising from the use of a mixture of Co^2+^ and Cd^2+^ sensitizers is the very broad range of suitable HCOOH concentrations over which PVG efficiency does not significantly change. This was demonstrated in the recent report on PVG of Ir,^34^ wherein the PVG efficiency ranged from 59 to 95% for 0.005–16 M HCOOH with only one indistinct maximum at 1 M HCOOH but no second sharp maximum at low concentrations. This was also demonstrated in this study for Ru, for which the same range of concentrations with minimum changes in PVG efficiencies (15–29%) was observed and only indistinct maxima at 0.01 and 8 M HCOOH were evident.

Since a 0.005 M HCOONa medium provided significantly higher PVG efficiencies for Ru and Re than 0.01 M HCOOH (cf. Figures 1 and 2), the impact of the most effective metal ion sensitizers (i.e., Cd^2+^ and Co^2+^) was also investigated in this medium at a sample flow rate 1.5 mL min^–1^. However, no positive effects were observed with either Cd^2+^ or Co^2+^ ions added into 0.005 M HCOONa for all three analytes, rather a strong negative effect or even a complete loss of the response with 1–10 mg L^–1^ Cd^2+^ and 5–50 mg L^–1^ Co^2+^. The effectiveness of Cd^2+^ and Co^2+^ thus seems to be strongly related to the acidity of the dilute medium.

### Impact on Other Analytes

Earlier reports concerning efficient PVG from dilute photochemical media did not address transition metals but were devoted to PVG of halides when employing a low concentration of Cu acetate (0.6–1.1 mM) as the sensitizer/photochemical medium^19^ and, very recently, to the PVG of As^3+/5+^ from dilute HCOONa (1 mM) in a sulfite medium.^47^

Attempts were thus made with other analytes (viz., Fe^3+^, Co^2+^, Ni^2+^, As^3+^, Se^4+^, Mo^6+^, Rh^3+^, Te^4+^, W^6+^, and Bi^3+^) to identify those exhibiting characteristics similar to those of Ru, Re, and Ir. Within the concentration range of 0.001–10 M HCOOH and at a sample flow rate of 2 mL min^–1^, PVG efficiencies were poor or not evident for W, Fe, and Rh, ultimately providing maximum values of around 0.001, 0.13, and 0.2%, respectively, from 10 M HCOOH, in agreement with previous studies.^10,23,36,37^ Tungsten and Rh require the presence of sensitizers (Cd^2+^ and Cu^2+^, respectively) that mediate the PVG process,^10,23^ while the PVG of Fe is significantly enhanced by adjusting pH to 2.0–3.0.^36,37^ No “second” maximum in the dependence of PVG efficiency of W on HCOOH concentration appeared in the presence of 100 mg L^–1^ Cd^2+^ as the sensitizer.

**Figure 3:**
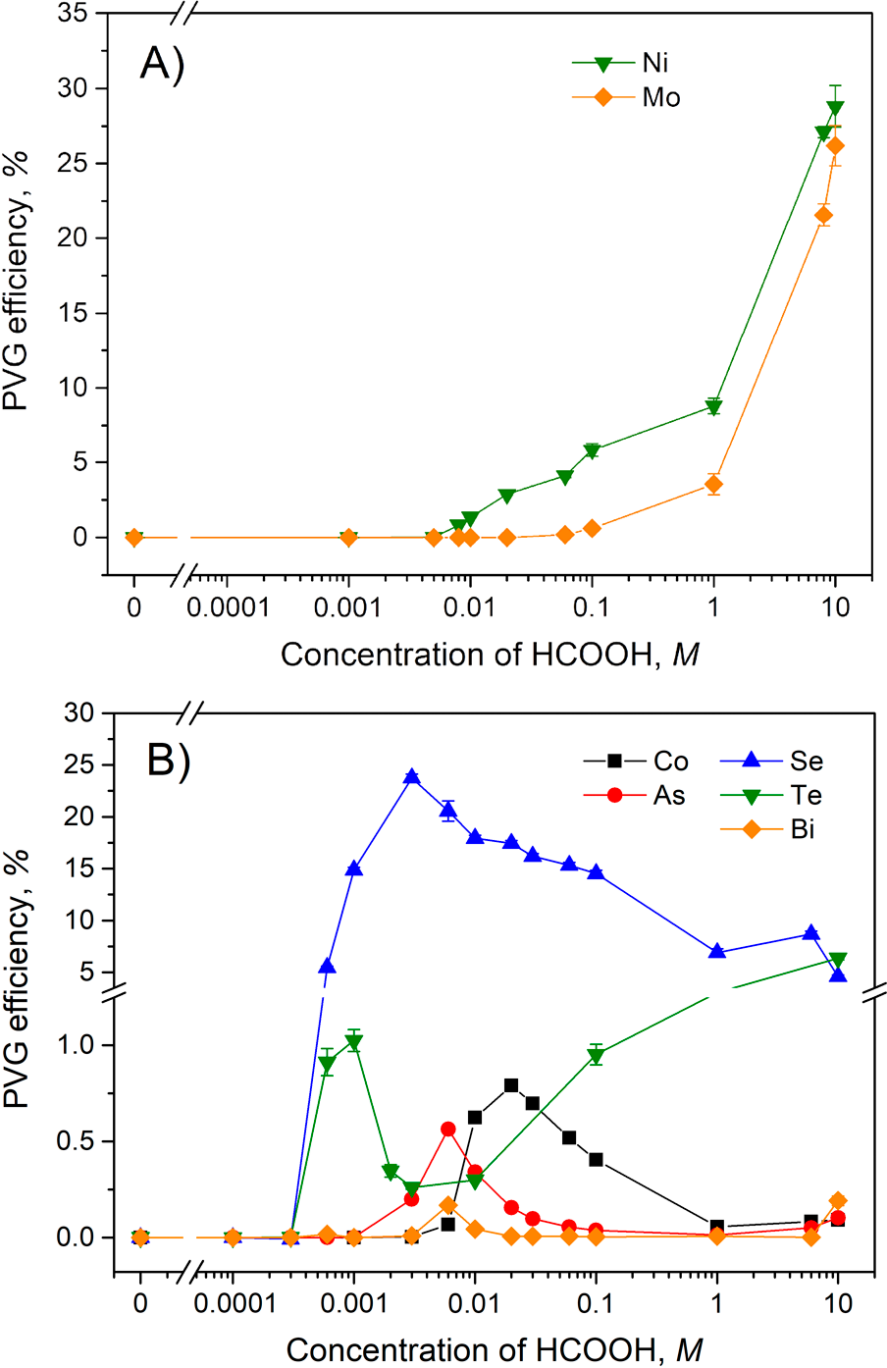
Influence of HCOOH concentration on PVG efficiency of (A) Ni and Mo and (B) Co, As, Se, Te, and Bi. Sample flow rate 2 mL min^–1^ (IT = 22 s); 2 μg L^–1^ Ni^2+^ and Mo^6+^, 5 μg L^–1^ Co^2+^, and 10 μg L^–1^ Se^4+^, As^3+^, Te^4+^, and Bi^3+^ examined for PVG. Data shown for Bi are multiplied 10-fold to better illustrate the effect. Note that the range of 0.0005 to 20 M is presented on a logarithmic scale.

For Ni and Mo, their PVG efficiency gradually increased with HCOOH concentration, characteristic of “normal” or expected behavior, as shown in Figure 3A. Volatile Ni(CO)_4_ and Mo(CO)_6_ are known to be easily generated from HCOOH media in the absence of sensitizers.^27,38,39,48^ Interestingly, the PVG of Ni is detected in HCOOH as low as 0.005 M, whereas an order of magnitude higher concentration (0.02–0.06 M) is required for Mo, illustrating that PVG from dilute HCOOH is analyte-specific and not a universal phenomenon.

Elements for which the PVG efficiency actually exhibits a maximum at low HCOOH concentrations and the 2 mL min^–1^ sample flow rate include Co^2+^, As^3+^, Se^4+^, Te^4+^, and Bi^3+^, as shown in Figure 3B, although the trends differ significantly. For Co, As, and Se, PVG efficiencies attained in the low range of HCOOH concentrations surpassed those at high concentrations, similar to those of Ru, Re, and Ir, but the HCOOH concentrations corresponding to the maxima differed slightly. As discussed above for Ir, this shift can, to some extent, arise as a consequence of differences in the photostability of the generated analyte species toward excessive UV irradiation. The highest PVG efficiency of 0.79% was attained using 0.02 M HCOOH for Co, while As, and possibly Bi, provided sharp local maxima at 0.006 M HCOOH, yielding PVG efficiencies of 0.56 and 0.017%, respectively. Tellurium exhibited a 1.0% PVG efficiency at 0.001 M HCOOH but higher efficiencies were obtained at HCOOH concentrations greater than 0.1 M. The behavior of Se differed in that relatively high maximum PVG efficiency of 24% was reached at 0.003 M HCOOH, thereafter slowly declining toward 1 M. This result is also reflected in the publication by Campanella et al.^49^ who irradiated samples in an FEP coiled reactor, partially permeable to 185 nm radiation, and reported a very sharp maximum at 0.3% HCOOH (i.e., around 0.065 M).

When CH_3_COOH or mixtures of HCOOH and CH_3_COOH comprise the photochemical medium instead of HCOOH, As, Se, and Bi generate no such significant “second” maximum in their dependences of PVG efficiency on concentration of acid(s) (see the Supporting Information).

### Effect of Dissolved Gases

Reductive conditions resulting from UV irradiation of photochemical media comprising low concentrations of HCOOH presumably generate volatile carbonyls of these elements, such as Ru(CO)_5_ or Ru_3_(CO)_12_,^31,33^ Re(CO)_5_H or Re_2_(CO)_10_, and Ir(CO)_4_H, Ir_2_(CO)_8_, or Ir_4_(CO)_12_.^34^ Highly reducing e_(aq)_^*–*^ and radical species (mainly H^•^ and CO_2_^•–^) are responsible for the reduction of analyte M^*n*+^ to M^0^, likely followed by the rapid uptake of cogenerated CO. The generation of CO by photolysis of HCOOH is thus critical to the formation of the volatile carbonyl species of these transition metals. The yield of CO was shown to be driven not only by the applied wavelength (185 nm vs 254 nm) and temperature, but even by addition of H_2_O_2_.^50^ Conversely, dissolved O_2_ was shown to inhibit production of CO during photolysis of HCOOH.^50^

With this in mind, the effect of dissolved CO in the photochemical medium on PVG of Ru, Re, and Ir was investigated initially without added sensitizers. Solutions of HCOOH (0, 0.005, and 0.01 M) and prepared standards were saturated with CO by bubbling a flow rate of 50 mL min^–1^ through the solution for 30 min at 20 °C prior to undertaking photolysis. Peak area responses originating from CO saturated standards of 40 μg L^–1^ Ru and Re and 0.1 μg L^–1^ Ir were compared to those obtained from standards without CO saturation. In parallel, the same standards and photochemical media were similarly saturated with Ar to eliminate dissolved O_2_ (as O_2_ is also removed during saturation with CO).

Saturation of DIW with CO (solubility of CO in DIW is 27.6 mg L^–1^ ≈ 0.001 M at 20 °C) permitted PVG of Ir at a sample flow rate of 2.0 mL min^–1^ (IT = 22 s) with an efficiency of about 1.0%. Such a significant effect of CO was not evident for Ru and Re at this sample flow rate, at which the measured signals were detectable but very low. Interestingly, increasing the sample flow rate to ≥4 mL min^–1^ (thus decreasing the IT!) led to significant enhancements in PVG efficiencies, reaching 0.047 and 5.0% for Ru and Ir, respectively, at 6 mL min^–1^ (IT = 7.2 s). Although maximum response for Re was attained at 4–5 mL min^–1^, the PVG efficiency remained very low (≈0.0002%). All signals from these experiments were confirmed to originate from the action of UV light because no response was evident with the same solution when the UV lamp was powered off.

A plausible explanation for the PVG of Ru, Re, and Ir from pure DIW saturated with CO involves reduction of Ru^3+^ to Ru^0^, Re^7+^ to Re^0^, and Ir^3+^ to Ir^0^ by e_(aq)_^*–*^ and/or H^•^ arising from simple homolysis of water (see below) by 185 nm radiation followed by uptake of the CO available from the saturated solution. Such a similar reduction-carbonylation is well known to occur for Ni during chemical vapor generation by NaBH_4_ in the presence of dissolved CO.^51,52^ Similar to Ru and Re, no PVG of Ni occurred from media saturated with CO and photolyzed at sample flow rates 2–3 mL min^–1^, whereas PVG was detected at 4 mL min^–1^ and efficiency gradually increased with increasing sample flow rates (lowering ITs) to approximately 2.5% at the highest tested sample flow rate of 7.5 mL min^–1^ (IT = 5.8 s). Conversely, no PVG of Mo from CO saturated solutions was achieved irrespective of the sample flow rate up to 7.5 mL min^–1^.

A clear positive effect of CO on PVG of Ru, Re, and Ir was also convincingly demonstrated by measurements at a “suboptimal” HCOOH concentration (0.005 M). Increases in PVG efficiencies of 2.7-, 5.2-, and 1.5-fold were obtained when solutions were saturated with CO compared to only 1.8-, 4.2-, and 1.2-fold increases from solutions deaerated with Ar. Positive effects of photolysis of 0.01 M HCOOH saturated with CO were not as evident if the impact of concurrent removal of dissolved O_2_ was compensated for. Solutions saturated with Ar typically provided higher responses than those simply saturated with CO. In the case of Ir, the peak area response was even lower compared to that of an unsaturated solution. This might be the result of the significant effect of dissolved O_2_ on PVG. In addition, dissolved CO may inhibit decomposition of HCOOH and scavenge generated e_(aq)_^*–*^ and free radicals as it may react with e_(aq)_^*–*^ to yield CO^•–^, thereby compromising reduction power.^53^

Dissolved O_2_ is a recognized quencher of radical-based reactions due to its affinity for e_(aq)_^–^ and H^•^.^1^ Its negative impact was clearly evident when solutions containing 0.01 M HCOOH were saturated with O_2_, leading to pronounced decreases in peak area response for all three elements, namely, 4.8-fold for Ru, 16-fold for Re, and 1.5-fold for Ir compared to those from nondeaerated solutions (containing room temperature saturation levels of dissolved O_2_). These data suggest that the PVG of Ru, Re, and Ir from dilute HCOOH media may not be robust and easily influenced by the presence of dissolved gases. Based on the substantial impact of O_2_, it may also be the reason for the much poorer repeatability and reproducibility of PVG efficiencies for Re from dilute HCOOH media, whereas the repeatability and reproducibility of the results for Ru, and especially Ir, were typically very good.

Finally, the impact of dissolved O_2_ on PVG was examined using a mixture of 10 mg L^–1^ Co^2+^ and 25 mg L^–1^ Cd^2+^ as sensitizers added to 0.01 M HCOOH with irradiation at a sample flow rate of 1.5 mL min^–1^ (optimal for Ru and Ir, see Table 1). No significant positive or negative effects (within ±6%) from dissolved CO, Ar, or O_2_ were evident for any of the three analytes, clearly suggesting a dramatic change in the mechanism by which PVG takes place in the presence of metal ion sensitizers.

### Supporting Experiments and Some Speculations on the Mechanism

In addition to the discussion presented above, an explanation for the positive effect of low concentrations of HCOOH compared to those conventionally optimized (1–20 M range) may be advanced based on the UV wavelength available for irradiation and its intensity, which is modulated by the absorption characteristics of the various media. To obtain further insights into the impact of UV wavelength on PVG of Ru, Re, and Ir from dilute HCOOH, the thin-film flow-through photoreactor was replaced with one comprising a common low-pressure 15 W germicidal mercury UV lamp whose circumference was wrapped with a 5.8 m length of 1 mm i.d. × 1.59 mm o.d. PTFE tubing (irradiated volume, 4.55 mL) through which the sample solution was propelled. This arrangement does not permit exposure of the analyte solution to 185 nm radiation.^7,49^ Moreover, the transmittance of 254 nm radiation is also significantly reduced when using a coiled PTFE reactor^54^ to approximately 52% of that of synthetic quartz for the ≈0.3 mm wall thickness tubing employed in this study. Keeping in mind that the inner quartz channels in the thin-film flow-through photoreactor are immersed in the low-pressure plasma and thus efficiently irradiated from all directions, substantially longer ITs (550–68 s) were utilized using the PTFE coiled reactor, corresponding to sample flow rates of 0.5–4 mL min^–1^, but no PVG response was detected from 40 μg L^–1^ Ru and Re and 1 μg L^–1^ Ir in 0.01 M HCOOH. Subsequently, the effect on PVG of Ru, Re, and Ir was investigated over a broad range of concentrations of HCOOH (0 and 0.001–10 M) and at sample flow rates of 2 and 4 mL min^–1^. At both flow rates, no second maximum appeared using dilute HCOOH. Some PVG action from Ru and Re was confirmed at ≥1 M HCOOH, whereas PVG of Ir was detected at 1 M HCOOH employing 4 mL min^–1^ and at 0.1 M employing 2 mL min^–1^. The largest PVG signals for Ru, Re, and Ir were expectedly recorded using 10 M HCOOH and, at the longer IT (i.e., at 2 mL min^–1^), efficiencies of ≈0.0015, 0.011, and 1.5%, respectively, were obtained, significantly lower than with the thin-film flow-through photoreactor.

In parallel with the highly efficient UV irradiation, the photochemical medium is also significantly heated when it passes through the thin-film flow-through photoreactor, which may affect the release of volatile species and PVG efficiency. Further experiments were thus focused on the effect of the sample flow rate on the temperature of various photochemical media at the outlet from the thin-film flow-through photoreactor. The thermocouple was inserted into the T-piece replacing the PTFE tubing typically introducing carrier Ar (see Figure S1). Two sample flow rates were tested (1 and 2 mL min^–1^) because the latter value provided the highest PVG efficiencies using an 0.01 M HCOOH medium while with the former sample flow rate the PVG efficiency was significantly attenuated (see Figure S3), attributed to the photodecomposition of generated volatile species due to excessive 185 nm irradiation. Only slight increases in temperature of the exiting photochemical media were evident; namely, the temperatures at 1 mL min^–1^ were 60.5 ± 1.0 °C, 60.5 ± 1.0 °C, and 61.5 ± 1.0 °C for DIW, 0.01 M HCOOH, and 8 M HCOOH, respectively, while at 2 mL min^–1^, the temperatures were 53.1 ± 1.0 °C, 53.5 ± 1.0 °C, and 55.5 ± 1.0 °C, respectively. It is obvious that the increase in temperature by around 6–7 °C for all the tested media is not significant and likely cannot be detrimental to the volatile species. This is supported by previous reports^34,43^ in which the measured trends of analytical responses on sample flow rates using 6 M HCOOH for Re and 10–14 M HCOOH for Ir were opposite because the response gradually increased with lower sample flow rates, even down to 0.5 mL min^–1^. All of these results thus suggest that the 185 nm radiation available in the thin-film flow-through photoreactor is responsible for the observed enhanced response in dilute HCOOH media.

The molar absorption coefficients of H_2_O and HCOOH at 185 nm differ by 3 orders of magnitude (0.0324 M^–1^ cm^–1^ for H_2_O and 34.9 M^–1^ cm^–1^ for HCOOH).^55^ Calculated penetration depths (attenuation of 185 nm radiation to 10%) at ambient temperature correspond to 0.56 cm in DIW, 0.47 cm in 0.01 M HCOOH, 0.19 cm in 0.1 M HCOOH, 0.027 cm in 1 M HCOOH, and 0.0029 cm in 10 M HCOOH. It is clear that in the low HCOOH concentration ranges (<0.001 M), 185 nm radiation is almost completely attenuated by only H_2_O,^56^ resulting in its homolysis (1) and (charge transfer to solvent) photochemical ionization (2):
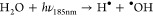
1
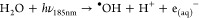
2wherein the quantum yield for reaction 2 is almost 1 order of magnitude lower.^57,58^ In the presence of increasing HCOOH concentrations, the following reactions become relevant:
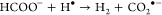
3
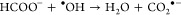
4wherein ^•^OH can react with both dissociated HCOO^–^ and undissociated HCOOH to form CO_2_^•–^ and HCO_2_^•–^, respectively.^58^ Moreover, photolysis of HCOOH by 185 nm and also 254 nm radiation yields H^•^, CO_2_^•–^ and e_(aq)_^–^ by several pathways.^1,2^ At low pH values arising from the presence of HCOOH, the reaction of e_(aq)_^–^ with H^+^ to form H^•^ must also be considered.

The standard reduction potentials of −2.9, – 2.3, and −1.9 V (vs SHE) for e_(aq)_^–^_,_ H^•^, and CO_2_^•–^ are all more than sufficient to enable complete reduction of the metal ion analytes investigated. As demonstrated in the previous section, wherein the feasibility of PVG of Ru, Re, and Ir from pure water saturated with CO was evident, it appears that sequential reduction of Ru^3+^ to Ru^0^, Re^7+^ to Re^0^, and Ir^3+^ to Ir^0^ occurs by H^•^ and/or e_(aq)_^*–*^ arising primarily from homolysis of water at 185 nm followed by rapid uptake of the available dissolved CO. Such reduction/carbonylation in water has also been demonstrated for Ni.

Experiments with HCOONa in the photochemical media affecting pH, which finally led to substantially higher PVG efficiencies for Ru and Re obtained with neat 0.005 M HCOONa, suggest an enhanced role for e_(aq)_^–^ in the reduction of Ru^3+^ to Ru^0^ and Re^7+^ to Re^0^ as e_(aq)_^–^ can be rapidly scavenged by H^+^ and converted to H^•^ at low pH. Conversely, PVG of Ir may be favored by the presence of H^•^ because it likely leads to generation of volatile Ir carbonyl hydride (e.g., Ir(CO)_4_H) as the most probable species.^59^ In a similar vein, the generation of gaseous CO by direct photolysis of HCOOH is crucial for the production of volatile Ru, Re, and Ir carbonyls. As evident from the work by Zechner and Getoff,^56^ the quantum yield of CO for the photolysis of HCOOH by 185 nm radiation gradually increases at ≥0.001 M HCOOH, while the yield of e_(aq)_^–^ significantly decreases at ≥0.0001 M HCOOH. In a 0.01 M HCOOH medium, a reasonable balance between the concentration of e_(aq)_^–^ required for efficient reduction and generated CO required for subsequent synthesis of metal carbonyls may exist. At higher HCOOH concentrations, UV photolysis of HCOOH leading to formation of CO_2_^•–^ may dominate processes relevant to homolysis of H_2_O, but CO_2_^•–^ may not have such reducing properties, or the reduction kinetics are slower. In addition, the amount of generated CO may not increase with HCOOH concentration, as demonstrated by the work by Adams and Hart.^50^ When PVG is conducted in the presence of metal sensitizers, the reaction mechanism is likely different and the action of CO_2_^•–^ may dominate, as evidenced by electron paramagnetic resonance (EPR) spin trapping techniques.^16,30,32,48^ Subsequently, the effect of dissolved O_2_ becomes irrelevant.

As evidenced from several experiments, an important aspect arises relating to the instability of the generated volatile species when exposed to excess UV irradiation (at 185 nm) because the penetration depth in dilute media is greater, leading to their more significant decomposition via decarbonylation reactions.^60,61^ This effect was also demonstrated in a medium of pure DIW saturated with CO from which PVG of Ni, Ru, Re, and Ir was significantly enhanced at higher sample flow rates (shorter ITs). The UV shadowing effect becomes significant at higher HCOOH concentrations, partially protecting the generated volatile species from decomposition. This effect may not fully explain the occurrence of the second maximum in PVG efficiency of Ru, Re, and Ir (and others) observed during optimization of the HCOOH concentration. No such second maximum occurs when PVG of Ni and Mo is conducted at substantially higher sample flow rates (4 and 6 mL min^–1^), resulting in ITs of 10.8 and 7.2 s, respectively. Again, the courses of dependences were very similar to those described in Figure 3A for the sample flow rate of 2 mL min^–1^, although the PVG efficiencies were significantly but expectedly lower due to insufficient UV irradiation.

## Conclusions

Compared to typical photochemical media that contain single to tens of molarity of HCOOH, repeatable and relatively efficient PVG from dilute HCOOH and HCOONa media (≈0.01 M) is achieved for Ru, Re, and Ir. The specific concentrations at which maximum PVG efficiency is obtained appear to be dependent on the applied IT, as evident with Ir, due to a UV shadowing effect and low stability of the generated volatile metal carbonyls toward excessive UV irradiation. The significant enhancement effect of added sensitizers, especially Cd^2+^ and Co^2+^, was confirmed as being present in such dilute HCOOH media, wherein the range of useful HCOOH concentrations characterized by high PVG efficiency becomes very broad, typically from 0.01 to 10 M HCOOH. Analysts pursuing the use of PVG must thus be cognizant of the potential benefits of examining very dilute HCOOH media, especially when the PVG of new analytes is investigated, or when a significant enhancement effect of added metal sensitizer(s) is identified.

Lower concentrations of HCOOH may definitely offer several advantages for analytical methodologies based on PVG, including lower consumption of reagents (attractive from the perspective of greener methodologies), lower risk of analyte contamination conjointly with lower limits of detection at subppt levels (see the Supporting Information for the analytical figures of merit achieved with 0.01 M HCOOH medium in the presence of 10 mg L^–1^ Cd^2+^ as a sensitizer), less dilution of real (water) samples with added volume of concentrated HCOOH required to reach optimum concentration,^38^ and reduced load of organic vapors on atomic detection systems when significant impact is evident. Conversely, dilute HCOOH media appear less tolerant toward dissolved gases and interferents in the liquid phase (see the Supporting Information and Figures S7 and S8 for the comparison of the effect of HNO_3_ on PVG of Ru, Re, and Ir conducted from 0.01 and 8 M HCOOH and 0.01 M HCOOH in the presence of 10 mg L^–1^ Cd^2+^) due to the likely reduction of e_(aq)_^*–*^ and free radical concentrations, which may limit real analytical applications. This aspect requires further investigation with individual analytes and sample matrices.

Nevertheless, the observation that volatile species of these metals can be generated from almost pure water is extremely interesting, especially from a mechanistic point of view. Other techniques are needed to help clarify this phenomenon, including EPR spin trapping to provide more insights into the presence of generated e_(aq)_^*–*^ and free radicals in such media.^16,30,32,48^ Convincing identification of the generated volatile species from dilute HCOOH media should also be undertaken; otherwise, plausible explanations of this phenomenon cannot be further advanced.

The range of elements for which similar behavior applies was expanded to include Co, As, Se, Te, and Bi and, in light of the recent report^47^ on PVG of As^3+/5+^ (as a hydride) achieved using highly dilute HCOONa media in the presence of sulfite, other such studies describing this phenomenon are likely to follow.

## Supporting Information Available

Specification of analytical standards and metal ion sensitizers, details on instrumentation including a scheme of a PVG arrangement coupled to ICPMS, typical ICPMS parameters for coupling with PVG, comparison of PVG transient signals from Ru, Re, and Ir simultaneously generated from 0.01 M HCOOH or DIW, influence of sample flow rate using 0.01 M HCOOH, experiments dealing with the reduction capabilities of dilute HCOOH medium and the effect of dilute CH_3_COOH, influence of the HCOONa fraction in the photochemical medium, influence of sample flow rate using 0.005 M HCOONa, experiments dealing with the impact of metal ion sensitizers, effect of CH_3_COOH on PVG of As, Se, and Bi, experiments dealing with testing of tolerance of PVG conditions toward HNO_3_ and determination of analytical figures of merit (PDF)
