# Supplementary material for: Efficient Photochemical Vapor Generation from Low Concentration Formic Acid Media
Source: Anal Chem. 2024 Jan 6;96(3):1241–50. doi: 10.1021/acs.analchem.3c04472 (PMC10809224; doi:10.1021/acs.analchem.3c04472)
Supplement: Supplementary file 1 — ac3c04472_si_001.pdf [file ac3c04472_si_001.pdf]

Supporting Information

Efficient Photochemical Vapor Generation from Low Concentration Formic Acid Media

Eva Jeníková,[a,b] Jaromír Vyhnanovský,[a,b] Karolína Hašlová,[a,b] Ralph E. Sturgeon[c] and Stanislav Musil[a,*]

[a] Institute of Analytical Chemistry of the Czech Academy of Sciences, Veveří 97, 602 00 Brno, Czech Republic

[b] Charles University, Faculty of Science, Hlavova 8, 128 43 Prague, Czech Republic

[c] National Research Council of Canada, 1200 Montreal Road, Ottawa, Ontario K1A 0R6, Canada

* Corresponding author; E-mail: stanomusil@biomed.cas.cz (S. Musil)

TABLE OF CONTENTS

Specification of analytical standards and metal ion sensitizers.. S-3

Details on instrumentation.. S-3

Figure S1. PVG arrangement for FI coupled to ICPMS with simultaneous liquid nebulization.. S-4

Table S1. Typical ICPMS parameters for coupling with PVG... S-5

Figure S2A–C. Comparison of PVG transient signals from Ru, Re, and Ir simultaneously generated from 0.01 M HCOOH or DIW.. S-6

Figure S3. Influence of sample flow rate using 0.01 M HCOOH as the photochemical medium on PVG efficiency of Ru, Re, and Ir.. S-7

Experiments dealing with the reduction capabilities of dilute HCOOH medium and the effect of dilute CH_3COOH.. S-7

Figure S4. Influence of HCOONa fraction in the photochemical medium on PVG efficiency of Ru, Re, and Ir.. S-8

Figure S5. Influence of sample flow rate using 0.005 M HCOONa as the photochemical medium on PVG efficiency of Ru, Re, and Ir.. S-8

Experiments dealing with the impact of metal ion sensitizers... S-9

Figure S6A–C. Effect of metal ion sensitizers on PVG efficiency of Ru, Re, and Ir from 0.01 M HCOOH... S-10

Effect of CH_3COOH on PVG of As, Se, and Bi investigated in the range 0.001–10 M and various mixtures of CH_3COOH and HCOOH on PVG of As, Se, and Bi keeping the total acid molarity of 0.006 M... S-11

Experiments dealing with testing of tolerance of PVG conditions towards HNO_3 and determination of analytical figures of merit.. S-11

Figure S7. Relative effects of added HNO_3 on PVG of Ru, Re, and Ir from either 0.01 M or 8 M HCOOH... S-12

Figure S8. Relative effects of added HNO_3 on PVG of Ru, Re, and Ir from 0.01 M HCOOH containing 10 mg L^{-1} Cd^{2+} as sensitizer.. S-13

References... S-15

EXPERIMENTAL SECTION

Analytical Standards and Metal Ion Sensitizers. Commercial stock analytical standard solutions of 1000 mg L^{-1} of individual elements were sourced as follows: Ru^{3+} (as $RuCl_3$) in 1 M HCl, Ir^{3+} (as $IrCl_3$) in 10% (m/v) HCl, and Bi^{3+} in 0.5 M HNO_3 were obtained from Sigma-Aldrich; Ni^{2+} in 2% (v/v) HCl, Rh^{3+} (as $(NH_4)_3RhCl_6$) in 5% (v/v) HCl, W^{6+} (as $(NH_4)_{10}W_{12}O_{41}.5H_2O$) in 2% (v/v) NH_4OH, Te^{4+} in 20% (v/v) HCl, and Re^{7+} (as NH_4ReO_4) in deionized water (DIW) from Analytika (Czech Republic); Mo^{6+} (as $(NH_4)_2MoO_4$) in 0.5% (m/v) NH_4OH from Absolute Standards, Inc. (USA); Co^{2+} in 1 M HCl and Fe^{3+} in 1 M HCl from BDH (UK); aqueous solutions of As^{3+} and Se^{4+} were prepared by dissolving sodium arsenite(III) and sodium selenite(IV) pentahydrate, respectively, (Lachema, Czech Republic) in DIW. Standard solutions containing Ir^{3+} and Ir^{4+} were also prepared by dissolving solid ammonium hexachloroiridate(III) ($(NH_4)_3[IrCl_6]$, Fluka) and ammonium hexachloroiridate(IV) ($(NH_4)_2[IrCl_6]$, Fluka) in 10% (m/v) HCl.

The following compounds were used as potential metal ion sensitizers for PVG: cadmium(II) acetate dihydrate (p.a., Lach-Ner), cobalt(II) acetate tetrahydrate (99.999%, Alfa Aesar), copper(II) acetate monohydrate (Merck), iron(II) acetate (≥99.99%, Sigma-Aldrich), manganese(II) acetate tetrahydrate (p.a., Sigma-Aldrich) and nickel(II) acetate tetrahydrate (p.a., Sigma-Aldrich). Stock solutions of metal sensitizers were prepared by dissolution of these metal acetates in 0.2% (v/v) CH_3COOH and contained 2.5–10 g L^{-1} of individual metals.

Instrumentation. Standard solutions were introduced into a stream of the photochemical medium with the aid of an injection valve (0.5 mL sample volume). Delivery at an arbitrary flow rate to the photoreactor was undertaken using a peristaltic pump (Reglo Digital, Ismatec) which was also used to evacuate waste from the gas-liquid separator (GLS). All connecting tubing was made of PTFE (i.d. 1 mm) with the exception of the Tygon pump tubing. The thin-film flow-through photoreactor was a 19W low-pressure mercury discharge lamp (Jitian Instruments Co., Beijing, China) internally fitted with three efficiently irradiated lengths of synthetic quartz tubing (total volume ≈0.72 mL) as well as two short quartz segments on either end of the photoreactor (≈0.25 mL) which serve as exterior inlet and outlet sample connection ports. These segments are not efficiently irradiated and are thus not taken into consideration when irradiation times are calculated. The effluent stream was mixed with a flow of Ar carrier and directed to a plastic GLS made from a polypropylene centrifuge vial of 15 mL internal volume. A 400 mL min^{-1} flow of Ar carrier was used to provide efficient release of generated

volatile species and their transport from the GLS to the ICPMS. The outlet of the GLS was connected via PTFE tubing (2 mm i.d. × 40 cm long) to an Agilent 8900 ICPMS/MS via an ultra-high matrix introduction (UHMI) port located downstream of a Scott double-pass spray chamber. Carrier liquid (2% (m/v) HNO_3) was mixed with an internal standard (IS) solution prepared in 2% (m/v) HNO_3 and was concurrently introduced into the spray chamber via a MicroMist nebulizer (Burgener Research Inc., Mississauga, Canada). The liquid carrier channel was equipped with a manual injection valve (0.5 mL sample loop volume) and this arrangement was exclusively utilized for estimation of overall PVG efficiency (see Section "Procedure, Data Evaluation, and Conventions").

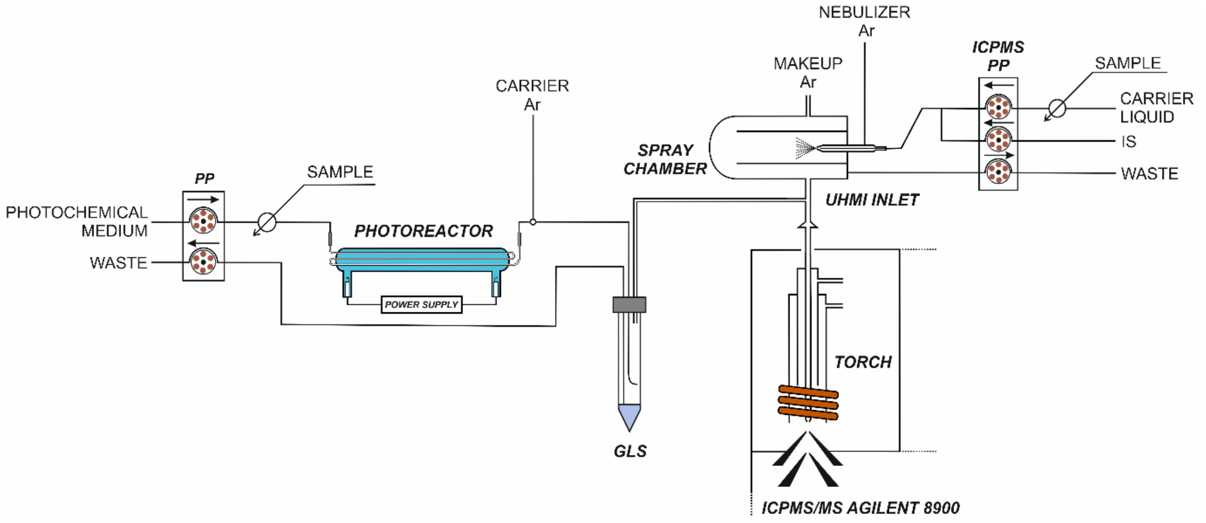

Figure S1. PVG arrangement for FI coupling to ICPMS with simultaneous liquid nebulization. GLS – gas-liquid separator, UHMI – ultra-high matrix introduction port, PP – peristaltic pump.

No special cleaning of the thin-film flow-through photoreactor was necessary between sequential measurements and the chemifold was typically only flushed with DIW at the end of the measurement day. From time to time, the quartz photoreactor was manually filled with concentrated HNO_3 (or reverse aqua regia) via a syringe and the UV lamp was powered on to initiate decomposition of acid(s) and to facilitate dissolution and removal of any deposited (metal) impurities.

In some experiments, the thin-film flow-through photoreactor was replaced by a coiled PTFE reactor based on a 15W germicidal mercury UV lamp (low-pressure, Cole-Parmer, USA), whose circumference was wrapped with a PTFE tubing through which the sample solution was propelled. The irradiated length of the PTFE tubing (1 mm i.d. × 1.59 mm o.d., Vici Jour Research, Switzerland) was 5.8 m, resulting in 4.55 mL irradiated volume.

Detection was achieved using an Agilent 8900 triple quadrupole ICPMS operating in time-resolved analysis and single quadrupole modes. Optimal plasma settings for PVG measurements and selected analyte and IS isotopes monitored are summarized in Table S1. A maximum of three analytes was monitored along with at least one IS selected on the basis of similar *m*/*z* and 1st ionization potential of the analytes during tests of PVG of other analytes from dilute media. Introduction of a He gas flow (4.1 mL min^{-1}) into the reaction/collision cell served to exclude eventual contributions from any polyatomic interferences to measured baseline intensity, e.g., $^{40}Ar^{16}O^+$ on $^{56}Fe^+$, $^{40}Ar^{18}O^1H^+$ on $^{59}Co^+$, $^{40}Ar^{38}Ar^+$ on $^{78}Se^+$, and particularly when high concentrations of metal ion sensitizers (in mg L^{-1}) were utilized for PVG, i.e., $^{40}Ar^{61}Ni^+$ and $^{38}Ar^{63}Cu^+$ on $^{101}Ru^+$). This approach was found essential when low PVG efficiencies of investigated analytes were encountered.

Table S1. ICPMS (Agilent 8900) parameters for coupling with PVG and measured isotopes of analytes and IS

RF power	1550 W
RF matching	1.4 V
Sampling depth	8.0 mm
Nebulizer Ar	860 mL min^{-1}
Makeup Ar	0 mL min^{-1}
Carrier Ar for PVG	400 mL min^{-1}
ICPMS peristaltic pump flow	0.1 rps (0.34 mL min^{-1} carrier liquid, 0.06 mL min^{-1} IS)
Spray chamber temperature	2 °C
Reaction/collision cell mode	He (4.1 mL min^{-1})
Acquisition mode	Time-resolved analysis
Scan type	Single quad
Measured isotopes (dwell time)[a]	^{101}Ru (0.1 s), ^{103}Rh (IS,[b] 0.05 s), ^{185}Re (0.1 s), and ^{193}Ir (0.1 s)

[a] other isotopes included: ^{56}Fe (0.1 s), ^{57}Fe (0.1 s), ^{59}Co (0.1 s), ^{60}Ni (0.1 s), ^{72}Ge (IS,[b] 0.05 s), ^{75}As (0.1 s), ^{77}Se (0.1 s), ^{78}Se (0.1 s), ^{95}Mo (0.1 s), ^{103}Rh (0.1 s), ^{115}In (IS,[b] 0.05 s), ^{125}Te (0.1 s), ^{133}Cs (0.1 s), ^{182}W (0.1 s), and ^{209}Bi (0.1 s); [b] employed as an internal standard

RESULTS AND DISCUSSION

PVG from Dilute HCOOH Media.

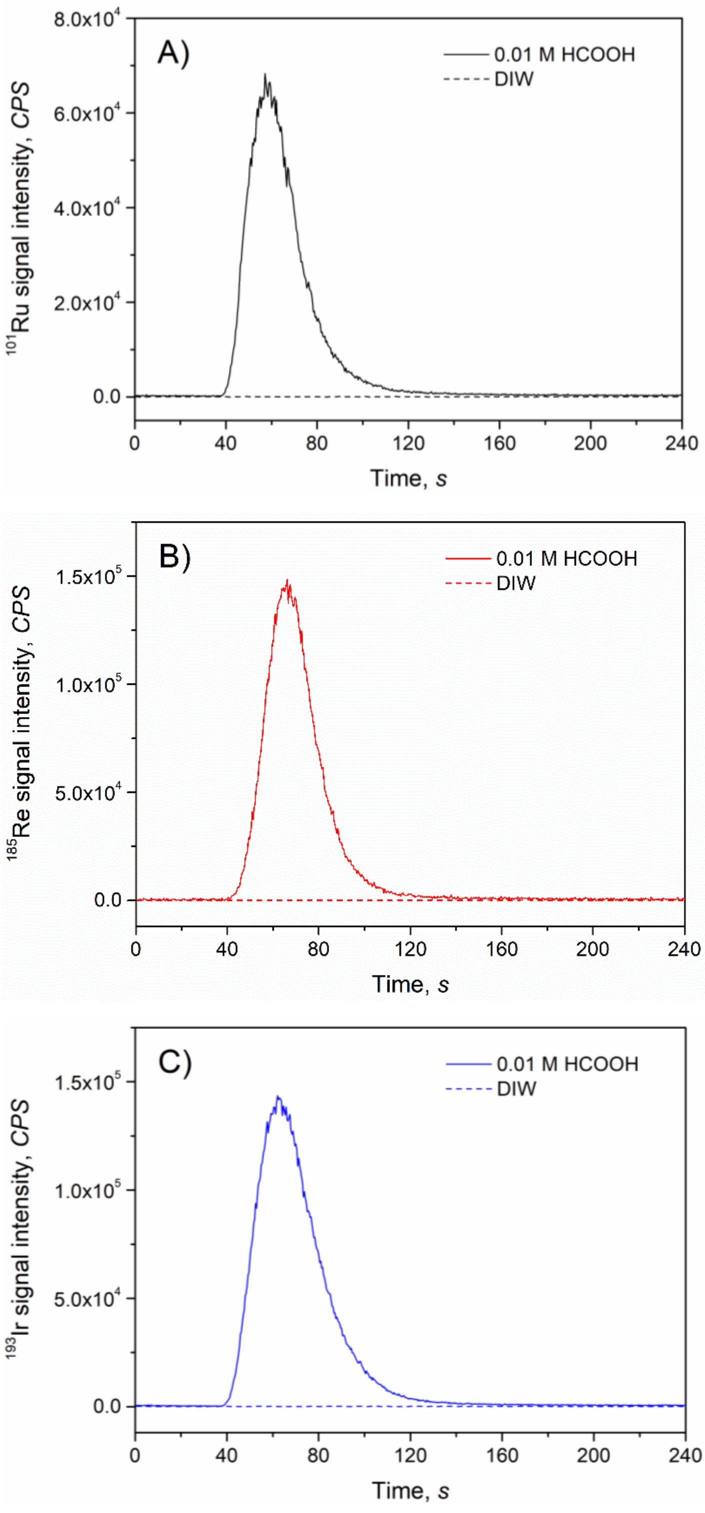

Figure S2. Comparison of PVG transient signals from A) 40 μg L^{-1} Ru, B) 40 μg L^{-1} Re, and C) 0.1 μg L^{-1} Ir simultaneously generated from 0.01 M HCOOH (solid line) or DIW (dashed line) as the photochemical media at a sample flow rate 2 mL min^{-1}.

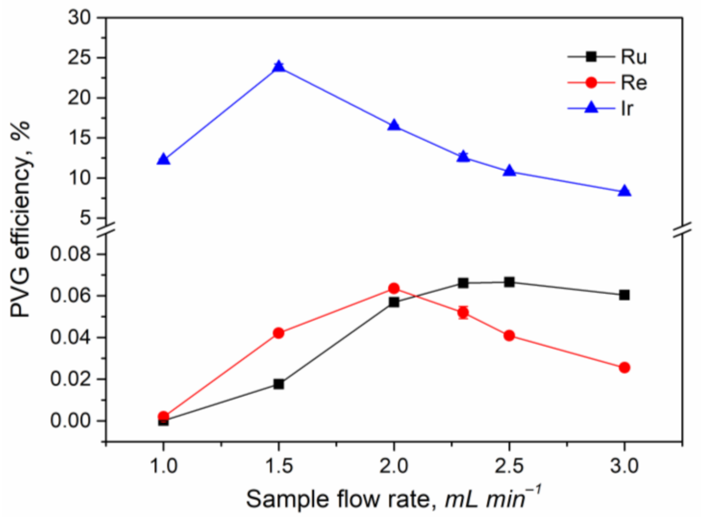

Figure S3. Influence of sample flow rate using 0.01 M HCOOH as the photochemical medium on PVG efficiency of Ru, Re, and Ir determined with 40 µg L^{-1} Ru and Re and 0.2 µg L^{-1} Ir. Uncertainties are represented as combined SD.

The reduction capabilities of the dilute HCOOH medium were also examined with different oxidation states of the elements. Ir^{3+} and Ir^{4+} were chosen for this purpose, employing standards prepared from solid hexachloroiridate(III) and hexachloroiridate(IV). No significant difference in peak area response was obtained with Ir^{3+} and Ir^{4+} standards when 0.01 M, 0.1 M, 1 M, and 10 M HCOOH were employed for PVG at a sample flow rate of 1.5 mL min^{-1}, hence, the reduction capability is sufficient to reduce both species to Ir^0.

Other dilute media were also examined for PVG of Ru, Re, and Ir. Absolutely no response was obtained for Ru and Ir using 0.005–1.0 M CH_3COOH while an 83-fold lower response was observed for Re at 0.01 M CH_3COOH in comparison to that measured using 0.01 M HCOOH. Addition of 0.001 M CH_3COOH to a 0.01 M HCOOH medium led to around 20% decrease in PVG efficiency for both Ru and Re but not for Ir. The decrease in PVG efficiency to around 50% of that achieved with neat 0.01 M HCOOH was identified at 0.005 M CH_3COOH for Ru, 0.01 M CH_3COOH for Re and 0.02 M CH_3COOH for Ir. To some extent, this may also be attributed to a sequestering of CO needed for synthesis of the volatile metal carbonyl species due to its reaction with $H_3C^{\bullet}$ to yield acetyl radicals,[1] altering the chemical synthesis of the desired products.

PVG from Dilute HCOONa Media.

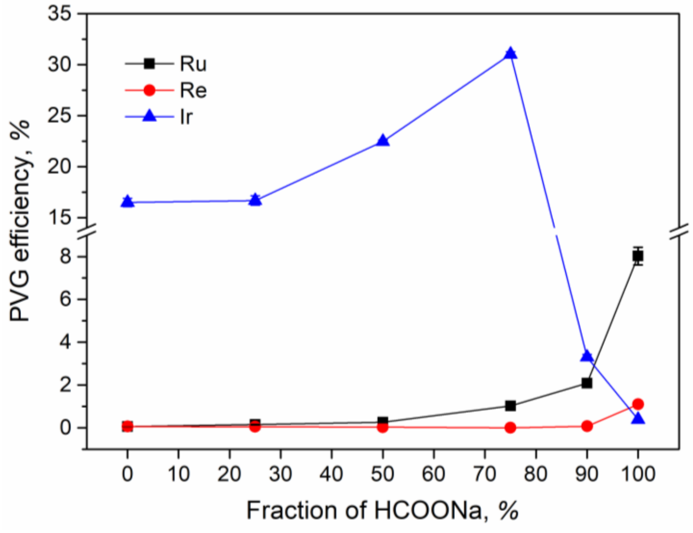

Figure S4. Influence of HCOONa fraction in the photochemical medium on PVG efficiency of Ru, Re, and Ir at a sample flow rate 2 mL min^{-1} (IT = 22 s). The sum of concentrations of HCOOH and HCOONa was always 0.01 M. 10 µg L^{-1} Ru and Re and 0.1 µg L^{-1} Ir were employed for PVG. Uncertainties are represented as combined SD.

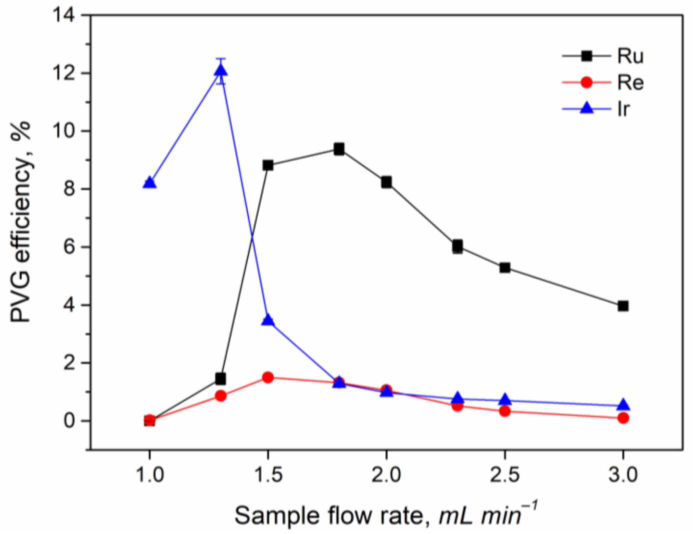

Figure S5. Influence of sample flow rate using 0.005 M HCOONa as the photochemical medium on PVG efficiency of Ru, Re, and Ir determined with 5 µg L^{-1} Ru and Re and 0.1 µg L^{-1} Ir. Uncertainties are represented as combined SD.

Impact of Metal Ion Sensitizers. Using a dilute HCOOH medium, the effect of the individual metal ion sensitizers on overall PVG efficiency of Ru, Re, and Ir was examined (Figures S6A–C). The metal ions (Cd^{2+}, Co^{2+}, Cu^{2+}, Fe^{2+}, Mn^{2+}, and Ni^{2+}) were chosen on the basis of their previously reported efficacy with PVG of various analytes[2,3] and were added only to the (mixed) analyte standards prepared in 0.01 M HCOOH and not to the carrier medium, which contained only 0.01 M HCOOH and into which flow the standard was injected.

The addition of 10–20 mg L^{-1} Cd^{2+}, 100–150 mg L^{-1} Co^{2+}, and 150–200 mg L^{-1} Fe^{2+} ions had the greatest positive effect on PVG of Ru, increasing the PVG efficiency by approximately 150-, 127-, and 115-fold, respectively (Figure S6A). The maximum enhancement was probably not reached with Co^{2+}, but higher Co^{2+} concentrations were avoided because peak area response measured with additions of 100 or 150 mg L^{-1} Co^{2+} was not significantly different. Similar impact was noted with added Fe^{2+} ions. Much lower, but still significant, enhancements were evident for Cu^{2+} in the range 1–5 mg L^{-1} (6.5–7.0-fold), for Mn^{2+} in the range 100–500 mg L^{-1} (2.6–8.0-fold), and Ni^{2+} at 50 mg L^{-1} (4-fold).

PVG of Re was impacted the most by the addition of Cd^{2+} ions, wherein a concentration of 5 mg L^{-1} Cd^{2+} increased the response by approximately 32-fold. In addition to Cd^{2+}, significant positive effects were identified for Fe^{2+} and possibly also for Co^{2+} ions, but these were much smaller than those arising with Cd^{2+}. Both Mn^{2+} and Ni^{2+} ions exhibited negative effects in the tested range of concentrations (10–500 mg L^{-1} for Mn^{2+} and 1–50 mg L^{-1} Ni^{2+}) as the response measured with the addition of sensitizers was always lower than that without added sensitizer (not evident in Figure S6B).

Cd^{2+} was the most effective sensitizer for PVG of Ir as well (Figure S6C), resulting in an approximately 4.6-fold enhancement at 5–10 mg L^{-1} Cd^{2+}. A smaller effect from Co^{2+} (around 2–2.2-fold) was evident over an extended range (5–100 mg L^{-1} Co^{2+}), while Fe^{2+} and Cu^{2+} ions exhibited a 1.4–1.6-fold increase at concentrations of 100–200 mg L^{-1} Fe^{2+} and 10 mg L^{-1} Cu^{2+}, respectively. No significant effects were associated with Mn^{2+} and Ni^{2+}.

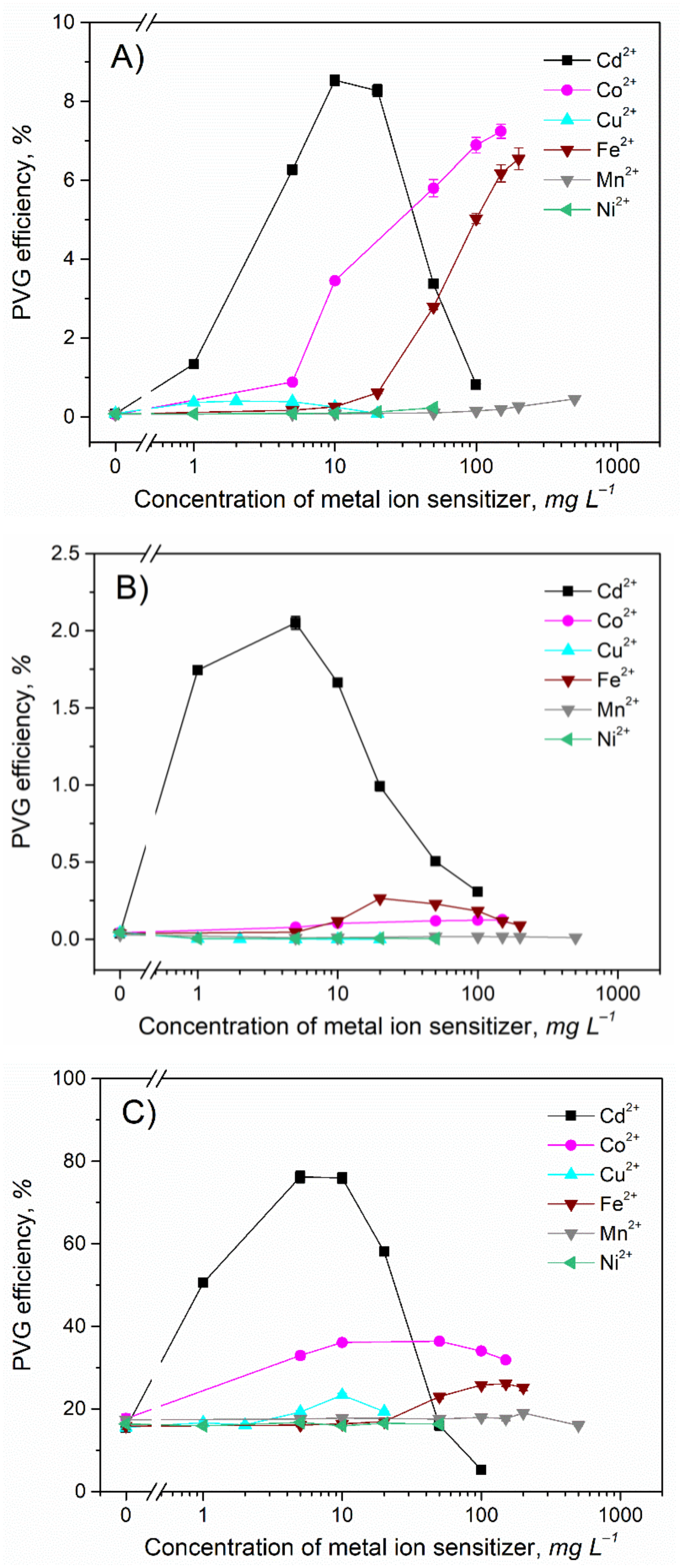

Figure S6. Effect of various metal ion concentrations on PVG efficiency of A) Ru, B) Re, and C) Ir using 0.01 M HCOOH and sample flow rate 2 mL min^{-1} (IT = 22 s). 10 µg L^{-1} Ru and Re and 0.1 µg L^{-1} Ir were employed for PVG. Uncertainties are represented as combined SD. The range 0.5 to 2000 mg L^{-1} is presented on a logarithmic scale.

Impact on Other Analytes. Since PVG of the hydride forming elements may be highly efficient from CH_3COOH based photochemical media, the effect of low concentrations of CH_3COOH on PVG of As, Se, and Bi was investigated in the range 0.001–10 M CH_3COOH. The observed dependences for As and Se differed significantly from those presented in Figure 3B and the PVG efficiencies gradually increased with CH_3COOH concentrations. An indistinct maximum was identified only for Bi at 0.02 M CH_3COOH but the PVG efficiency remained low (0.006%) and was comparable to that obtained with 10 M CH_3COOH. This is in accord with results from earlier studies that reported efficient PVG of Bi only from media comprising a mixture of HCOOH and CH_3COOH and in the presence of Fe^{3+} or Co^{2+} as sensitizers.[4-6] When a mixture of HCOOH and CH_3COOH was employed for PVG, keeping the total molarity of 0.006 M (based on the observed maxima of PVG efficiencies for As and Bi in Figure 3B), the PVG efficiency for Bi was enhanced from 0.017% (0.006 M HCOOH) by around 3-fold to 0.05% using 0.005 M HCOOH + 0.001 M CH_3COOH and also using 0.003 M HCOOH and 0.003 M CH_3COOH. Addition of ≥0.004 M CH_3COOH to the mixture significantly dropped the response to a level <15% of that obtained with 0.006 M HCOOH. There was no significant enhancement identified for As and Se using any combination of CH_3COOH and HCOOH (total molarity of 0.006 M), wherein the response even decreased by 55% and 20% for As and Se, respectively, using 0.003 M HCOOH + 0.003 M CH_3COOH and further gradually dropped at higher fractions of CH_3COOH.

Interferences and Analytical Practicality. When using dilute HCOOH media for analytical application, tolerance toward interferences may be compromised due to the lower concentrations of generated $H^{\bullet}$, $CO_2^{\bullet-}$, and $e_{(aq)}^{-}$ needed for analyte ion reduction. The significantly better tolerance towards HNO_3 as the most powerful scavenger of free radicals[7] was previously convincingly demonstrated when PVG of Ir was conducted with higher concentrations of HCOOH; however, this arose in the presence of Co^{2+} and Cd^{2+} sensitizers.[8] In this work, the tolerance towards HNO_3 was first compared without sensitizers, employing either 0.01 M HCOOH or 8 M HCOOH. In both cases, the same concentrations (i.e., 40 µg L^{-1} Ru and Re and 0.2 µg L^{-1} Ir) and a sample flow rate of 2 mL min^{-1} were used. The effects are summarized in Figure S7, from which it is clear that nitrate/nitrite (the latter arising from photolysis of nitrate) severely impact PVG efficiencies for all three analytes. PVG of Re was the most susceptible to the presence of HNO_3 with rather similar serious negative effects exhibited in both photochemical media. The difference in the effects of HNO_3 were not

particularly evident up to 0.1 mM HNO_3 for Ru and 1 mM HNO_3 for Ir (with the exception of the unclear decline in the recovery to 80–90% persisting in the range 0.01–1 mM HNO_3), while significantly better tolerance was obtained at concentrations ≥1 mM HNO_3 for Ru and ≥10 mM HNO_3 for Ir when an 8 M HCOOH photochemical medium was used. In addition to the potential scavenging of $e_{(aq)}^-$ and other free radicals, the effect of pH may play some role at the 10 mM level because pH decreases from 2.9 for neat 0.01 M HCOOH to around 2, thus also changing the degree of dissociation of HCOOH to $HCOO^-$. Moreover, NO_3^- has a relatively high absorption coefficient at 185 nm (around 5600 M^{-1} cm^{-1})[9] in comparison to undissociated HCOOH (around 55 M^{-1} cm^{-1}),[10] resulting in a significant UV shadowing effect and consequent lower rate of HCOOH photolysis.

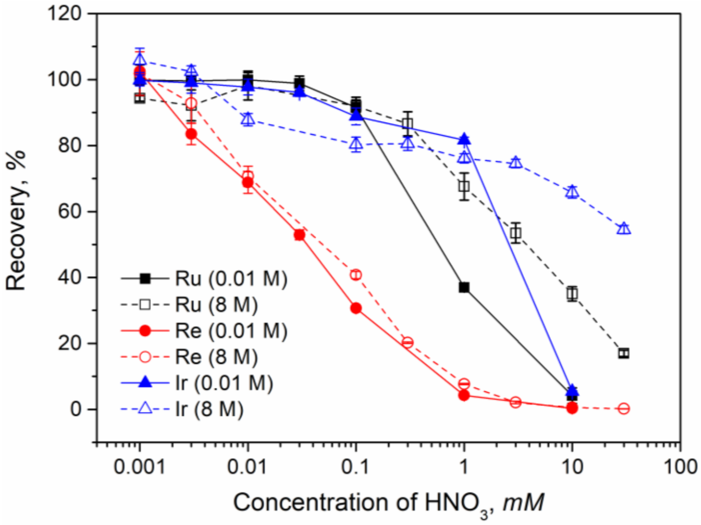

Figure S7. Relative effects of added HNO_3 on PVG from 40 μg L^{-1} Ru and Re and 0.2 μg L^{-1} Ir prepared in 0.01 M HCOOH (solid lines) and in 8 M HCOOH (dashed lines) acquired at a sample flow rate 2 mL min^{-1}. Uncertainties expressed as combined SD (n ≥ 3). HNO_3 concentration is presented on a logarithmic scale.

The tolerance of PVG of Ru, Re, and Ir towards HNO_3 was also examined when employing 0.01 M HCOOH containing 10 mg L^{-1} Cd^{2+} as the sensitizer and sample flow rate of 2 mL min^{-1}. These conditions were selected on the basis of fairly high PVG efficiencies for all three analytes (see Table 1 and Figures S6A–C). It is evident in Figure S8 that addition of 10 mg L^{-1} Cd^{2+} to 0.01 M HCOOH substantially increased the tolerance of PVG of Re towards HNO_3 (cf. Figure S7), by approximately 2 orders of magnitude in HNO_3 concentration, while the tolerance remained comparable for Ru or was even slightly worse for Ir. Interestingly, the significant interference (response attenuation by ≥10%) occurs at similar HNO_3 concentration (i.e., >0.3 mM) for all three analytes. Compared to the previous reports on PVG of Ru and Ir,[8,11]

when PVG was conducted from 8 M and 4 M HCOOH, respectively, and in the presence of 10 mg L^{-1} Co^{2+} and 25 mg L^{-1} Cd^{2+} as sensitizers, the tolerance limit for this dilute HCOOH medium occurs at an order of magnitude lower concentrations. All these results really suggest a worse tolerance of 0.01 M HCOOH media towards HNO_3 interference, even in the presence of metal ion sensitizers. In addition, this interference appears to be largely due to the NO_3^- anion itself and not only to a change in the pH of the reaction medium because the impact of added $NaNO_3$ at 1 mM was similar although slightly less negative, namely, the recoveries for 1 µg L^{-1} Ru, 1 µg L^{-1} Re and 50 ng L^{-1} Ir were 33 ± 1%, 47 ± 1% and 62 ± 2%, respectively (cf. Figure S8).

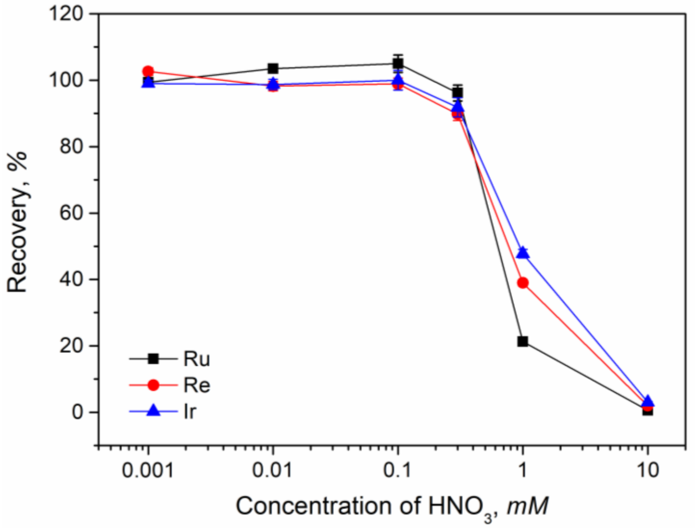

Figure S8. Relative effects of added HNO_3 on PVG from 1 µg L^{-1} Ru and Re and 50 ng L^{-1} Ir prepared in 0.01 M HCOOH containing 10 mg L^{-1} Cd^{2+} as sensitizer acquired at a sample flow rate 2 mL min^{-1}. Uncertainties expressed as combined SD (n ≥ 3). HNO_3 concentration is presented on a logarithmic scale.

To present an example of analytical practicality of the PVG-ICPMS methodology, the limits of detection (LOD) employing a dilute HCOOH medium were estimated. As in the case of testing HNO_3 interference, the PVG conditions employing 0.01 M HCOOH with 10 mg L^{-1} Cd^{2+} as sensitizer and sample flow rate 2 mL min^{-1} were selected. The LODs were evaluated by a repeated measurement (3σ, n = 11) of a blank solution (0.01 M HCOOH containing 10 mg L^{-1} Cd^{2+}) and the sensitivities were, for this purpose, derived from the linear calibration functions ($R^2 \geq 0.9997$) constructed using 0, 10, 50, and 100 ng L^{-1} Ru and Re and 0, 1, 5, and 10 ng L^{-1} Ir standards. The resulting LODs for simultaneous measurement of ^{101}Ru, ^{185}Re, and ^{193}Ir isotopes were outstanding, namely, 210 pg L^{-1} (105 fg absolute) for Ru, 60 pg L^{-1} (30 fg absolute) for Re, and 3.4 pg L^{-1} (1.7 fg absolute) for Ir. (Note: the linear dependence of the

peak area response, and thus also PVG efficiency, was identified up to at least 50 μg L^{-1} Ru, Re, and Ir although such high concentration levels were not employed during LOD evaluation to avoid longer wash-out of the analytes from the PVG system).

These LODs can be compared to those recently achieved in our laboratory using similar settings of ICPMS (measured isotopes and reaction cell mode) but with PVG conditions employing high HCOOH concentrations and Co^{2+} and Cd^{2+} sensitizers (the conditions listed in Table 1 with resulting PVG efficiencies). The LOD for Ru in this study is 5-fold higher than that previously reported,[11] which is, correspondingly, in line with the difference in PVG efficiency (8.5% vs. 29%, see Table 1). For Ir, the difference in PVG efficiency is not serious (76% vs. 89%)[8] and the LOD obtained with the dilute HCOOH medium is almost 2-fold lower. This decrease in LOD may be attributed to decrease in analyte contamination because the reported LOD was driven by the variance of the peak area of the blank[8] that corresponded to approximately 0.015−0.03 ng L^{-1} Ir while absolutely no persistent peak was detected in this work using a dilute HCOOH medium. For Re, we cannot provide such a direct comparison because the investigation of PVG of Re from high HCOOH concentrations has not yet been completed. We can only compare it to the LOD of 1 ng L^{-1} reported by Zhen et al.,[2] which is approximately 17-fold higher than that achieved with the dilute HCOOH medium. However, the fact that different ICPMS instrumentation was employed may influence this comparison.

REFERENCES

(1) Porter, G. B.; Benson, S. W. The Reaction of Carbon Monoxide with Free Radicals. *J. Am. Chem. Soc.* **1953**, *75*, 2773–2774.

(2) Zhen, Y.; Chen, H.; Zhang, M.; Hu, J.; Hou, X. Cadmium and cobalt ions enhanced-photochemical vapor generation for determination of trace rhenium by ICP-MS. *Appl. Spectrosc. Rev.* **2022**, *57*, 318–337.

(3) Jeníková, E.; Nováková, E.; Hraníček, J.; Musil, S. Ultra-sensitive speciation analysis of tellurium by manganese and iron assisted photochemical vapor generation coupled to ICP-MS/MS. *Anal. Chim. Acta* **2022**, *1201*, No. 339634.

(4) Vyhnanovský, J.; Yildiz, D.; Štádlerová, B.; Musil, S. Efficient photochemical vapor generation of bismuth using a coiled Teflon reactor: Effect of metal sensitizers and analytical performance with flame-in-gas-shield atomizer and atomic fluorescence spectrometry. *Microchem. J.* **2021**, *164*, No. 105997.

(5) Yu, Y.; Jia, Y.; Shi, Z.; Chen, Y.; Ni, S.; Wang, R.; Tang, Y.; Gao, Y. Enhanced Photochemical Vapor Generation for the Determination of Bismuth by Inductively Coupled Plasma Mass Spectrometry. *Anal. Chem.* **2018**, *90*, 13557–13563.

(6) Yu, Y.; Zhao, Q.; Bao, H.; Mou, Q.; Shi, Z.; Chen, Y.; Gao, Y. Determination of Trace Bismuth in Environmental Waters by ICP-MS with Cobalt Ion-Assisted Photochemical Vapour Generation. *Geostand. Geoanal. Res.* **2020**, *44*, 617–627.

(7) Lopes, G. S.; Sturgeon, R. E.; Grinberg, P.; Pagliano, E. Evaluation of approaches to the abatement of nitrate interference with photochemical vapor generation. *J. Anal. At. Spectrom.* **2017**, *32*, 2378–2390.

(8) Musil, S.; Jeníková, E.; Vyhnanovský, J.; Sturgeon, R. E. Highly Efficient Photochemical Vapor Generation for Sensitive Determination of Iridium by Inductively Coupled Plasma Mass Spectrometry. *Anal. Chem.* **2023**, *95*, 3694–3702.

(9) Duca, C.; Imoberdorf, G.; Mohseni, M. Effects of inorganics on the degradation of micropollutants with vacuum UV (VUV) advanced oxidation. *J. Environ. Sci. Health A* **2017**, *52*, 524–532.

(10) Weeks, J. L.; Meaburn, G. M. A. C.; Gordon, S. Absorption Coefficients of Liquid Water and Aqueous Solutions in the Far Ultraviolet. *Radiat. Res.* **1963**, *19*, 559–567.

(11) Musil, S.; Vyhnanovský, J.; Sturgeon, R. E. Ultrasensitive Detection of Ruthenium by Coupling Cobalt and Cadmium Ion-Assisted Photochemical Vapor Generation to Inductively Coupled Plasma Mass Spectrometry. *Anal. Chem.* **2021**, *93*, 16543–16551.
